# Impact of 3.0 T Cardiac MR Imaging Using Dual-Source Parallel Radiofrequency Transmission with Patient-Adaptive B1 Shimming

**DOI:** 10.1371/journal.pone.0066946

**Published:** 2013-06-18

**Authors:** Haipeng Jia, Cuiyan Wang, Guangbin Wang, Lei Qu, Weibo Chen, Queenie Chan, Bin Zhao

**Affiliations:** 1 Shandong Medical Imaging Research Institute, Shandong University, Jinan, People’s Republic of China; 2 MR Research Collaboration, Philips. Ltd. China, Shanghai, China; University of Adelaide, Australia

## Abstract

**Objectives:**

To prospectively evaluate the impact of 3.0 T Cardiac MR imaging using dual-source parallel radiofrequency (RF) transmission with patient-adaptive B1 shimming compared with single-source RF transmission in the RF homogeneity, image contrast and image quality.

**Methods:**

The study was approved by the local institutional review board, and all subjects provided written informed consent. Fourteen healthy volunteers were examined at 3.0 T MR, with both the conventional single-source and the new dual-source RF transmission. B1 calibrations (RF shimming) of the heart region were performed to acquire a percent of the prescribed flip angle (FA) of B1 maps, which were used for quantitative assessment of RF homogeneity. Contrast ratios (CRs) between ventricular blood pool and septum were calculated on balanced-turbo field echo (B-TFE) cine images. The off-resonance artifacts of cine images were blindly assessed by two radiologists according to a 4-point grading-scale.

**Results:**

A significantly lower mean coefficients of variance of the achieved FA with dual-source revealed better RF homogeneity compared to single-source (*P = *0.0094). Dual-source RF shimming significantly increased the CRs (*P*<0.05) and reduced the off-resonance artifacts of B-TFE cine images (*P*<0.05). Inter-observer agreement for the off-resonance artifacts of B-TFE cine images was good to excellent (*k* >0.65).

**Conclusions:**

Dual-source parallel RF transmission significantly improves the RF homogeneity, increases image contrast and reduces image artifacts of cardiac B-TFE images compared to single-source mode. This may be of value in reducing the observer-dependence of cardiac MR images and enhancing diagnostic confidence for clinical practice using CMR at 3.0 T.

## Introduction

In recent years, higher-field strength MR systems (≥3.0 T) have been more and more used in both clinical diagnosis and scientific researches by using the balanced steady-state free precession (b-SSFP) imaging, such as cardiac function, cardiac flow analysis and the other imaging techniques [1]–[3]. However, high-field MR imaging at 3.0 T also comes with some technical issues, including radiofrequency (RF) field inhomogeneity, local specific energy absorption rate (SAR) peaks and susceptibility artifacts [1]. The increase in RF power deposition and the presence of off-resonance artifacts are closely related to the nonuniformity of the main magnetic field (B0) and RF field (B1) [1], [4]. B0 field uniformity is critical at 3.0 T, while maintaining the homogeneity of the B1 field is also more of a challenge. The uniformity of the RF field is associated with a homogeneous distribution of the flip angle (FA) across imaging volume. This factor allows for an optimized SAR distribution and reducing local SAR peaks, which makes it possible at a high-field MR to lower the repetition time (TR) or increase FA of the SSFP sequences [1], [5]–[7]. The minimum TR achievable for SSFP sequences at 3.0 T potentially facilitates a shortening of the total acquisition time [5], [8]. Additionally, B1 inhomogeneity in turn increases its susceptibility to B0 inhomogeneity.

Multi-channel parallel RF transmission system has been proposed as a means for improving B1 homogeneity [9]–[11]. Increasing the number of RF transmit channels adds extra degrees of freedom which can be used to benefit B1 uniformity improvement and local SAR peaks reduction for body imaging at 3.0 T [12]. Dual-source RF transmission technique is designed to enable independently control phase and amplitude of each of the two RF waveforms by using two independent RF transmit channels. With this setup and full software control, it allows for calibration of the RF pulse and thus enables local RF shimming. Dual-source parallel RF transmission with patient-adaptive B1 shimming was first used and evaluated in abdomen, pelvis and spine imaging, which was proved to be able to improve B1 uniformity, reduce dielectric shading and accelerate imaging [5], [13]. Recently, several studies have reported that the implementation of dual-source RF excitation allows for an improved SAR model (shorter TR) and improved B1 homogeneity in heart, leading to improved image quality of cine images in the academic conference for MR in medicine [14]–[16]. However, a systematic clinical study of RF shimming in cardiac imaging was rare. Until recently, a relevant study firstly revealed the advantages of dual-source parallel RF transmission over conventional single-source RF transmission for cardiac imaging [17]. However, the small sample size, single-center-based applications, the lack of evaluation of changes in TR or FA to image quality are important limitations of most of the repots [14]–[17].

Due to its susceptibility to B1 inhomogeneity in the cardiac balanced-turbo field echo (B-TFE) cine sequence [8], [14], this study was to prospectively assess the impact of dual-source parallel RF transmission with patient-adaptive B1 shimming in the local RF homogeneity, image contrast and artifacts occurrence for cardiac MR imaging using B-TFE sequence at 3.0 T.

## Materials and Methods

### Ethics Statement

The study protocol was conducted with the approval of the local institutional review board of Shandong Medical Imaging Research Institute, Shandong University. Informed written consent was obtained from all participating volunteers before their enrollment in the study.

### Subjects

Fourteen healthy volunteers without history of cardiovascular disease (stable angina, or atypical chest pain) were consecutively enrolled in a prospective study. Inclusion criteria for participation in this study were ability to offer written informed consent and age no less than 18 years. Exclusion criteria were contraindications to MR imaging (severe claustrophobia, metallic implants such as cerebral aneurysm clips, ocular metallic deposits).

### CMR Imaging Protocol

Cardiac MR imaging were performed on a 3.0 T MR scanner (Philips Healthcare, Best, Netherlands) equipped with flexible dual-source parallel RF transmission system (Multi-Tx, protocols: maximum achievable gradient amplitude, 80 mT/m; rise time, 0.2 msec; slew rate, 200 T/m/sec). Images were acquired once with Multi-Tx switched on (dual-source), and once with Multi-Tx switched off (single-source). Two 16-channels phased-array coils integrated were used for signal reception. All MR imaging acquisitions were ECG gated.

B1 Map imaging: For each subject, a cardiac cine imaging was performed after the B1 calibration scanning. The B1 calibration image of the short-axis plane across the heart was acquired utilizing a saturation-recovery, dual-flip angle method with a segmented echo-planar imaging read out [18].The scanning parameters were used as follow: TR/TE, 1000/5.7 msec; dual flip angle (FA), 60°/120°; saturation delay, 500 msec. Based on the information of B1 calibration, the amplitude and phase of RF transmit channels were set and revised for subsequent sequences imaging with RF shimming within the local region of interest (ROI). In addition, the RF shimming parameters were partly influenced by the user-defined local ROI (individual difference in the heart region), yielding patient-adaptive local RF shimming for cardiac imaging [17]. The B1 calibration imaging generated the B1 map of short-axis plane during end diastole in a single breath hold, with dual-source and single-source models for direct comparison.

B-TFE imaging: A cardiac cine acquisition of short-axis plane perpendicular to the inter-ventricular septum with breath-holding was performed with parameters: 6 slices from the ventricular base to the apex; slice thickness, 8 mm without slice gap; FOV, 320×320 mm; matrix, 200×256; number of heart phases, 30; TR was chosen as short as the system allowed with maximal readout bandwidth (with and without dual-source mode); echo time (TE) was set to half of TR. In order to compare the effect in MR images acquisition between the dual-source and single-source, with a special focus on the revised TR or FA in the dual-source transmit system, four groups of parameters were designed to compare with each other. Group M0 was with single-source RF excitation, and with typical parameters: shortest TR/TE, 3.4/1.7 ms; FA, 45°. Groups M1, M2 and M3 were utilizing dual-source excitation with local B1 shimming. The parameters of group M1 were the same as group M0; FA increased from 45° to the maximum allowable FA 58° in group M2 with TR/TE kept to 3.4/1.7 ms; TR/TE values reduced to the shortest TR/TE 2.8/1.4 ms in group M3 with FA kept to 45°. Each volunteer was imaged according to the above 4 groups of parameters described. The CMR imaging parameters were summarized in Table 1.

**Table 1:** Imaging parameters of the B-TFE sequence for short-axis ventricular cine.

Group	RF	TR (ms)	TE (ms)	FA (°)
**M0**	Single-source	3.4	1.7	45
**M1**	Dual-source	3.4	1.7	45
**M2**	Dual-source	3.4	1.7	58
**M3**	Dual-source	2.8	1.4	45

Notes: *TR* repetition time, *TE* echo time, *FA* flip angle.

### Image Post-processing and Analysis

**Figure 1:**
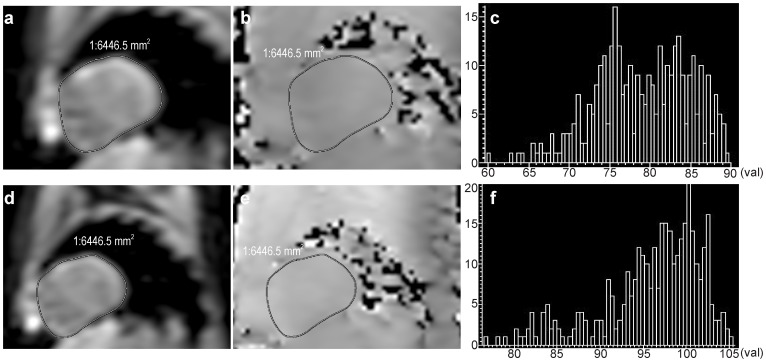
RF homogeneity data acquisition and image post-processing. Short axis cardiac modulus images (a, d) and B1 maps (b, e) acquired with single-source (a, b) and dual-source (d, e) RF excitation. The ROIs outlining the heart were placed on the modulus images (a, d), and then the same ROIs were copied to B1 maps (b, e) to evaluate the B1 homogeneity. Improved B1 homogeneity and reduced areas of dark spots were seen on (e) compared with (b). The distribution of the mean percentage of the achieved FA calculated from all pixels inside the ROI of the B1 maps was shown on figure c (without) and f (with dual-source RF excitation). The distribution of FA on (f) was more concentrated and uniform than that on (c). In addition, the average percentage of achieved FA on (f) was higher than that on (c).

RF homogeneity: The homogeneity of the RF field in the local volume was calculated from all pixels inside the ROI of the B1 map with and without dual-source excitation (exemplified with ROI analysis in Fig. 1a, b, d, e). A ROI outlining the heart was manually drawn on the modulus image and the same ROI was copied to corresponding B1 map. The pixel values in the B1 map were scaled as a percent of the prescribed (desired) FA. Then the mean percentage of the achieved FA (x) and standard deviation (ό) were calculated automatically on the workstation (EWS, Philips Healthcare). Coefficient of variance of the mean percentage of the achieved FA (CV = ό/x) within the ROI was used to evaluate RF homogeneity, where a lower ratio corresponded to more uniformity.

Image quality: Image quality evaluation for cardiac cine imaging included image contrast (CRs) and the occurrence of off-resonance artifacts. In each subject, CRs between blood pool and interventricular septum consisted of left ventricular (LV)-to-septum contrast and RV-to-septum contrast. A ROI covering an area of approximately 120 mm^2^ was manually drawn in the middle interventricular septum on the end-diastolic short-axis image. Then the same ROI was copied to the blood pool of LV and RV (avoiding papillary muscle) respectively. The mean signal intensity was obtained automatically. Each data was measured 3 times by the same reader, and the average of results was used finally. CR between blood pool and interventricular septum was calculated as follows [5], [19]:
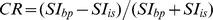
(SI_bp_ = signal intensity of blood pool and SI_is_ = SI of interventricular septum). CRs of 4 groups of cardiac cine images (M0–M3) were compared with each other.

The occurrence of off-resonance artifacts was assessed by two experienced radiologists independently on a 4-point grading-scale: 4–No off-resonance artifacts, excellent image quality; 3–Mild off-resonance artifacts, diagnostic but not perfect; 2–Moderate off-resonance artifacts, diagnostic on most segments but not all; 1–Serious off-resonance artifacts, not diagnostic.

### Statistical Analysis

Data analysis was performed using SPSS 17.0 (SPSS INC., Chicago, Illinois). A paired t-test was performed to compare RF homogeneity data with and without dual-source excitation. The calculated CRs of multiple groups were compared with one-way analysis of variance (ANOVA), followed by SNK (Student-Newman-keuls) test. The artifacts data were analyzed with non-parametric Wilcoxon signed rank test. Inter-observer agreement was evaluated with Cohen's kappa test. The strength of inter-observer agreement was defined as follows: 0.0–0.2, poor agreement; 0.21–0.4, fair agreement, 0.41–0.6, moderate agreement; 0.61–0.80, good agreement; and 0.81–1.0, excellent agreement. Statistical tests were two-tailed, and *P*<0.05 was considered to be significant.

## Results

### Subject Characteristics

All the fourteen healthy volunteers (9 men, 5 women; mean age, 45±17 years; range, 28–69 years) underwent CMR imaging successfully. The mean body mass index of the included volunteers was 25.7±3.9. All subjects showed regular heart rate and rhythm (HR, 67.5±11.3 bpm; rate range, 60–90 times per minute).

### RF Homogeneity

**Figure 2:**
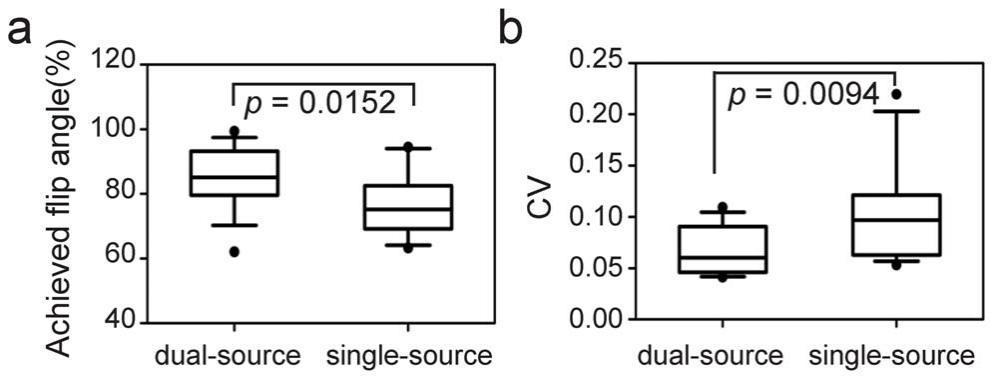
Quantitative assessment of RF field homogeneity. Box-type diagrams showed average percentage of achieved FA (a) and coefficient variance (CV) (b) as a measurement of RF homogeneity for dual-source and single-source. A higher average percentage of FA and a lower mean CV was obtained by using of dual-source mode. Both parameters improved with dual-source from all subjects.

A representative image demonstrating the benefit of local volume RF shimming using the dual-source RF excitation was shown in Fig. 1. For the mean percentage of achieved FA, the dual-source mode was significantly higher than that of single-source mode (85.38% ±9.46 and 76.74% ±9.7, *P* = 0.0152). The mean CV of dual-source mode and single-source mode were 0.066±0.022 and 0.103±0.05 respectively, with statistically significant difference (*P* = 0.0094). The quantitative analysis of the B1 maps was summarized in Fig. 2.

### Image Quality

**Figure 3:**
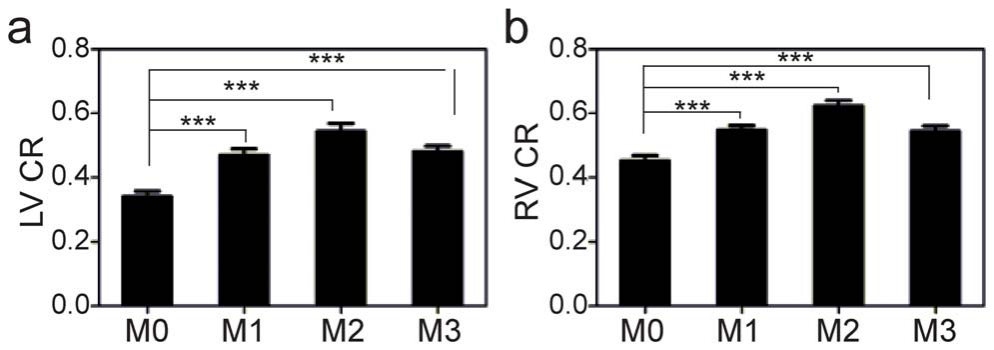
Image contrast at B-TFE cine imaging. Bar graphs showed CRs at LV-to-septum contrast (a) and RV-to-septum contrast (b) imaging with and without dual-source RF shimming. For CRs, the images with use of dual-source RF shimming (M1, M2, M3) revealed greater LV-to-septum and RV-to-septum versus the corresponding images with single-source mode. The CR of M2 was the largest with the FA = 58°. *** P<0.001 vs. single-source mode.

The CRs of group M1, M2 and M3 were all significantly higher than that of group M0 both in LV-to-septum and RV-to-septum (*P1*<0.001, *P2*<0.001, *P3* = 0.001 for LV-CRs, and *P1*<0.001, *P2*<0.001, *P3*<0.001 for RV-CRs, respectively). Within the dual-source mode, the CR of group M2 was the largest with significant difference (M2 vs M1, *P* = 0.0014 for LV and *P* = 0.0117 for RV; M2 vs M3, *P* = 0.0009 for LV and *P* = 0.0315 for RV), while there was no significant difference between group M1 and M3 (*P* = 0.874 for LV and *P* = 0.689 for RV). Comparisons of LV and RV CRs between the four groups were summarized in Table 2 and illustrated in Fig. 3.

**Table 2:** Comparison of the LV(RV)-to-septum contrast within the four groups.

	Group	Mean	SD	95% CI	P values
**LV**					
	**M0**	0.452	0.059	0.418–0.486	1.0
	**M1**	0.548	0.054	0.516–0.579	<0.001
	**M2**	0.624	0.061	0.588–0.659	<0.001
	**M3**	0.544	0.062	0.508–0.580	0.001
**RV**					
	**M0**	0.341	0.063	0.304–0.377	1.0
	**M1**	0.469	0.074	0.426–0.512	<0.001
	**M2**	0.543	0.093	0.489–0.597	<0.001
	**M3**	0.481	0.066	0.442–0.519	<0.001

Notes: The *P* value was comparison between group M0 and groups M1, M2, M3, separately.

**Figure 4:**
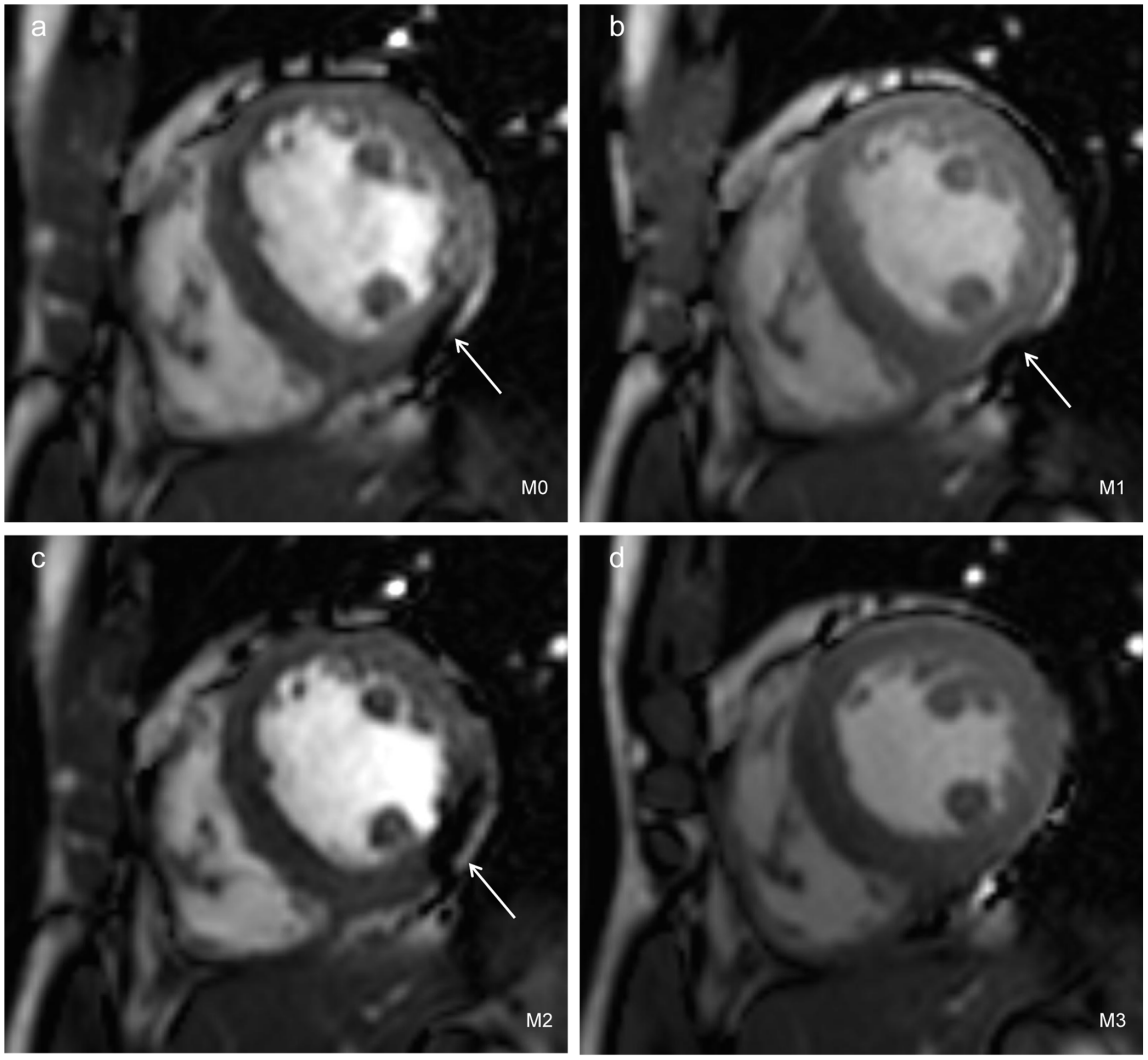
Artifacts on the short-axis B-TFE images of four groups. The cine images acquired in groups M0 (a), M1 (b) and M2 (c) were all suffered from dark banding artifacts (thin arrows) seriously. Compared with group M0 (a), group M1 (b) reduced the dark banding artifacts to some extent and was better on the image homogeneity. Within the four groups, the CR of group M2 (c) was largest when the FA was increased, however, the off-resonance artifacts of group M2 (c) was pronounced in the ventricular wall (thin arrows). The off-resonance artifacts (thin arrows) of group M3 (d) were reduced and shifted away from the heart with dual-source RF shimming and a shortest TR, furthermore, group M3 (d) achieved good CR of blood-to myocardium contrast.

The artifacts scores of group M1, M2 and M3 were higher than that of group M0, while there was only statistical significance between group M3 and group M0 (M3 vs M0: reader A: 3.57±0.51 vs 2.14±0.36, *P* = 0.0002<0.05; reader B: 3.64±0.49 vs 2.28±0.46, *P* = 0.0001<0.05). The artifacts scores of group M3 were the highest among the three groups with dual-source mode (reader A: M3 vs M1, *P* = 0.0001; M3 vs M2, *P* = 0.0001. reader B: M3 vs M1, *P* = 0.0005; M3 vs M2, *P = *0.0005). The artifacts scores of the four groups were summarized in Table 3 and illustrated in Fig. 4.

**Table 3:** Comparison of the off-resonance artifacts scores within four groups.

Group	Reader A	Reader B	Weighted Kappa
**M0**	2.14±0.36	2.28±0.46	0.65
**M1**	2.36±0.49	2.71±0.72	0.72
**M2**	2.50±0.52	2.50±0.76	0.69
**M3**	3.57±0.51	3.64±0.49	0.85

Inter-observer agreement of the off-resonance artifacts of the B-TFE ventricular cine images was good to excellent by the two radiologists (*k = *0.65, 0.72, 0.69 and 0.85 for group M0, M1, M2 and M3, respectively (Table 3).

## Discussion

B1 field inhomogeneity on high-field-strength MR systems has been a pronounced technical issue of clinical applications and researches. B1 field inhomogeneity resulted in nonuniform distribution of FA in the heart region, which brought degradation of image quality and reduction in the blood-to-myocardium contrast. In this study, the new system of dual-source parallel RF transmission with patient-adaptive B1 shimming in the heart region significantly 1) improved the homogeneity of RF field, led to more uniform distribution of FA across the heart region and increased CRs of the blood-to-myocardium; 2) reduced the off-resonance artifacts of the heart and its surrounding tissues by using the shortest TR available with the use of dual-source mode.

Initial studies have reported several approaches on improving B1 field uniformity, such as post-processing methods or using dielectric cushions [20], [21]. Unfortunately, the above-mentioned methods did not obtain satisfactory results in every imaging area. With the development of local RF shimming, a fixed anatomy-dependent preset mode of RF shimming was used to improve B1 homogeneity [22], which was closely related to individual patient’s anatomy and position in the MR imaging system. In cardiac imaging, B1 uniformity of local RF field will be obviously affected by patient size, the sternum metal wire and pleural or pericardial effusion etc [13], [17]. Therefore, the traditional fixed local RF shimming mode couldn’t better meet the requirements for ensuring consistently high image quality in cardiac imaging.

Traditional single-source RF excitation, using a single transmit source with a 90° phase shifter, the single RF signal is transported to the two ports of a quadrature RF coil. Dual-source parallel RF excitation, two completely independent RF sources are delivered to independent ports of the system quadrature body coil by using two independent RF transmit channels with software control [13]. With this design, B1 homogeneity can be significantly improved [5], [13], [17].

The use of dual-source parallel RF excitation allows for calibration of the RF pulse (the shape of RF waveforms) and thus enables local B1 shimming, which can be clearly illustrated on B1 maps [13], [17]. In this study, the mean percentage of achieved FA of the dual-source parallel RF excitation was significantly higher than that of the single-source mode (*P* = 0.0152). Dual-source parallel RF excitation with patient-adaptive B1 shimming in the heart region would enable us to achieve a relatively higher FA, which was closer to the prescribed FA for cardiac imaging (85.38% with volume RF shimming vs 76.74% without). The mean CV of the percentage of the achieved FA decreased from 0.103±0.05 with single-source to 0.066±0.022 with dual-source patient-adaptive RF shimming (*P* = 0.0094), which revealed a more uniform RF field distribution. Therefore, the use of dual-source RF excitation is effective in getting a relatively higher, more uniform and consistent FA in the heart region, which will be conducive to improving the quality of cardiac MR imaging.

In addition, the more uniform B1 field and subsequently homogeneous FA distribution result in an optimized SAR distribution within the imaging volume, thereby reducing local SAR peaks. This makes it possible for high-field strength MR at 3.0 T to lower the TR (within the International Electrotechnical Commission limits) [13], [17]. Recent studies have demonstrated that the use of dual-source parallel RF shimming significantly improved B1 homogeneity, which allowed for increasing the prescribed FA and reducing TR at 3.0 T MR to improve image quality [1], [5].

B-TFE sequences could yield higher SNR and contrast between myocardium and blood than spoiled gradient-echo sequences [23], [24]. Therefore, B-TFE sequences have become the preferred sequences for assessing cine imaging associated with global cardiac function and cardiac flow assessment at 1.5 T [25]. For 3.0 T MR, B-TFE sequences are more susceptible to B1 inhomogeneity and non-uniform FA distribution, resulting in a reduction in image contrast, especially in the right ventricle and septum. Our study results showed that the use of dual-source RF excitation (M1, M2, M3) could achieve better CRs between blood pool and interventricular septum (biventricular image contrast) compared with single-source imaging (M0). Within the dual-source mode, the CRs of M2 was the largest when FA was increased from 45° to 58° compared with M1 and M3. Individualized B1 shimming with use of dual-source parallel RF excitation and increased FA significantly improved the blood-to-septum contrast. Thus, improved image contrast using dual-source RF shimming will provide better tissues depiction and may therefore offer more accurate measurements of cardiac function indexes, resulting in a higher diagnostic yield in future CMR imaging. There was no significant difference between the CRs of group M3 and that of group M1, which indicated that a certain extent of shortening TR for B-TFE sequence did not significantly affect the image contrast between the tissues. Furthermore, depending on the subject’s heart rate, reduced TR of the B-TFE has the added benefits of accelerating the acquisition speed per heart phase or reducing the breath-hold duration, which may be propitious to patients with severe disease for cardiac MR imaging [5], [17]. In our study, the acquisition of B-TFE sequence with a higher number of phase-encoding steps (30 phases), this gain of the time saved would be even larger.

For 3.0 T MR, B1 inhomogeneity also induces B-TFE amplified off-resonance artifacts, decreasing image quality and limiting its clinical use in cardiac imaging [8]. In this study, the gain in CRs of group M2 was the largest; however, the cardiac cine images at M2 suffered from more dark banding artifacts compared with group M3 with a lower TR. Therefore, an increase in the prescribed FA may in turn make B-TFE imaging more susceptible to B1 inhomogeneity, referring to inconsistent FA distribution in imaging volume [14], [26], [27]. There are two main strategies to reduce banding artifacts in B-TFE imaging: reduce the B0 and B1 field inhomogeneities across the heart, and reduce the TR [8]. Previous studies suggested localized linear or higher order local shimming procedures and phase-specific shimming correction with cardiac triggering improved B0 homogeneity, resulting in consistent off-resonance artifacts suppression [6], [28]. Conventional single-source MR systems mainly employed frequency scouting and shifting to reduce B1 field inhomogeneity induced artifacts in B-TFE imaging [8]. In this study, we adopted linear B0 shimming in the cardiac cycle. Our results demonstrated that dual-source parallel RF excitation significantly reduced the off-resonance artifacts at 3.0 T cardiac MR imaging compared to single-source mode. The off-resonance artifacts of group M3 were significantly reduced and were scored highest with a shortest TR. Thus, the use of dual-source parallel RF transmission with patient-adaptive B1 shimming and a further reduction in TR may significantly decrease the susceptibility to off-resonance artifacts and improve image quality in future cardiac cine imaging. Inter-observer agreement for the off-resonance artifacts of the B-TFE ventricular cine images with and without dual-source RF shimming was good to excellent by the two radiologists (*k* >0.65), preferably dual-source RF shimming. The gains in imaging quality of B-TFE with dual-source RF transmission are conducive to reduce the observer-dependence on cardiac MR images post-processing [29], [30], and may be of value in increasing the reproducible consistency and the diagnostic confidence for routine usage of CMR at 3.0 T.

### Conclusions

In conclusion, dual-source parallel RF transmission with patient-adaptive RF shimming can significantly improve B1 field homogeneity and increase contrast of cardiac B-TFE cine images. An improved B1 homogeneity with a shortest TR can also help to reduce image artifacts substantially and accelerate imaging speed. These gains in acquisition with the dual-source parallel RF transmission have shown us a wide application prospects in clinical CMR at 3.0 T.
